# Uncertainty quantification on a spatial Markov-chain model for the progression of skin cancer

**DOI:** 10.1007/s00285-019-01367-y

**Published:** 2019-12-19

**Authors:** Fred Vermolen, Ilkka Pölönen

**Affiliations:** 1 Delft Institute of Applied Mathematics, Delft University of Technology, Delft, The Netherlands; 2 Faculty of Information Technology, University of Jyväskulä, 40014 Jyvaskyla, Finland

**Keywords:** 60J20, 65C05, 65C40

## Abstract

A spatial Markov-chain model is formulated for the progression of skin cancer. The model is based on the division of the computational domain into nodal points, that can be in a binary state: either in ‘cancer state’ or in ‘non-cancer state’. The model assigns probabilities for the non-reversible transition from ‘non-cancer’ state to the ‘cancer state’ that depend on the states of the neighbouring nodes. The likelihood of transition further depends on the life burden intensity of the UV-rays that the skin is exposed to. The probabilistic nature of the process and the uncertainty in the input data is assessed by the use of Monte Carlo simulations. A good fit between experiments on mice and our model has been obtained.

## Introduction

Ageing of populations in industrialised society puts a huge burden on health care. In particular, next to cardio-vascular diseases, cancer is one of the most lethal diseases developing in these societies. Reasons are a sedentary lifestyle, diets that contain large amounts of sugar, as well as bad habits such as smoking.

An underestimated class of cancers concerns skin cancer. According to the WHO (2017), one in every three cancers diagnosed is classified as a skin cancer. There are three major types of skin cancers including basal-cell carcinomas (BCC), squamous-cell carcinomas (SCC) and different types of melanoma. Each of these cancers develop in different cells: BCC develops in basal cells which are squamous cells located close to the basement membrane, and metastasises rarely. SCC develops from squamous cells, and metastasises more often. Finally, melanomas, which develop from melanocytes, form the most dangerous class of skin cancers. Melanomas metastasise if they grow deeper and penetrate through the basal membrane. All these cancers are able to metastasise to other parts of the body.

Skin cancer is often caused by a long-term exposure of skin to UV-radiation, since UV-radiation increases the risk of skin cancer. People with lighter skin and who travel to the mountains, such as during skiing when the UV radiation is large at high altitudes, and further amplified by the reflection of snow, are subject to dangerous portions of radiation. It should also be noted that people, who expose their skins over a long period of time to strong doses of sunlight on the beach or in water, have a high risk of developing burns and skin cancer. According to Eriksson and Tinghög (2015), the total societal cost of skin cancer in Sweden over the year 2011 were estimated at 178 million Euros, with an increase of 27% with respect to 2005.

The current paper deals with a three dimensional spatial Markov chain model for the simulation of skin cancer in general. Mathematical modelling studies that engage themselves with skin cancer in particular are very scarce, and therefore we list some relevant mathematical studies of general solid tumors, although we admit that the list is not complete. Significant contributions to the simulation of metastasis of general cancer using Markov chain modelling and Monte Carlo simulations were realised by Newton et al. (2012, 2013, 2015). Elements from game theory were used in the form of the Prisoner’s Dilemma in West et al. (2016).

Next to these probabilistic approaches, several studies on cellular automata models applied to cancer have been performed. In this context, we mention a few important studies. Poleszczuk and Enderling (2013) considered a cellular automata model with probabilities of cell division and cell migration. They consider a two-dimensional lattice with points that are either occupied by a cancer cell or not. If the cancer cell proliferates, then it does not migrate in their model. They use constant values for the likelihoods for migration and proliferation. As far as we know, in the literature, the study by Poleszczuk and Enderling (2013) is closest to the current study. Poleszczuk and Enderling (2013) give a clear description of how their method works. Further, Butler et al. (2014) formulated a cellular-automaton model for general cancer growth in two dimensions, where processes like cell death (apoptosis) of cancer cells, immunity death and angiogenesis are incorporated. Their approach for cancer cell migration is similar to the study by Poleszczuk and Enderling (2013). Monteagudo and Santos (2012) present a cellular automaton model that was used by Butler et al. (2014). The latter group applied the model to predict the impact of drugs on cancer growth. Jiao and Torquato (2011) described a cellular automaton model for emergent behaviours regarding invasive tumor growth in heterogeneous tissues. Their model is more complicated and contains interactions between several cell types, cell division, degradation of extracellular matrix (ECM), mutation rates and cell mobilities. Alarcon et al. (2003) also consider tumor growth in inhomogeneous environments, in which the oxygen concentration is computed, and where blood vessels are assumed to be immobile. Though the model is based on cellular automata principles, the nature of their model is entirely deterministic. Kansal et al. (2000) propose a cellular automata model for the simulation of brain tumors, where their domain is represented by Voronoi cells. Each Voronoi cell represents a biological cell that grows, divides or dies. By far one of the earliest cellular automaton models for cancer was developed by Qi et al. (1993), who consider cell migration, cytotoxic behaviour of the immune system and mechanical pressure of the tumor.

A different class of computational models is formed by the agent-based models. In these models, the time history of each cell is taken into account, and the position of the cells is, unlike in cellular automata models, continuous over time and only becomes discrete after application of a numerical method for the time-integration. Processes like cell migration, differentiation, proliferation and mutation of each cell are simulated using this class of models. Numerous examples from the literature exist for the development and application of agent-based models. Although the list is far from complete, we mention several studies in agent-based models that were applied to diseases in the (epi)dermis. Li et al. (2013) simulate epithelial renewal in skin over a three years period. Grabe and Neuber (2007) simulate the dynamics of psoriasis as a disease in the epidermis. Sun et al. (2009), Adra et al. (2010) develop a three-dimensional model for epidermal wound healing and structuring with the interplay of the TGF-beta growth factor. Thingnes et al. (2012) simulate the temporal evolution of the melanocyte distribution on the basis of (negative) chemotaxis due to a substance that is secreted by the keratinocytes. We also note our own work on agent-based models with, among others, an application to wound contraction as a result of serious burn trauma, see Boon et al. (2016).

We list three mathematical studies devoted to melanoma. The first study is due to Gallinaro et al. (2013) and treats a model for melanoma cell migration with an elastic continuum. The second study by Morais et al. (2017) treats a 2D probabilistic Widom-Rowlinson model that shares some features with the current model and with the model by Poleszczuk and Enderling (2013). The third study by Ciarletta et al. (2001) entails a partial differential equations (PDE) based approach for the radial progression of melanoma. We will elaborate more on these models in Sect. 5.

The current paper proposes a spatial Markov chain model that is entirely stochastic. The model divides the domain of computation into nodal points that have two possible states: either ‘cancer state’ or ‘not in cancer state’. The transition probability depends on the status of the neighbours in terms of distance and number of neighbours that are in ‘cancer state’. Next to this intrinsic likelihood, we propose an update of the transition probability as a function of the intensity of UV-radiation as well as the transmission of UV into the skin. As an innovation compared to the previously formulated cellular and Markov chain models for cancer development, the current model is simple in the sense that it consists of three input parameters only, and that the transmission probability of a nodal point depends on the number of neighbours that are in ‘cancer state’ and on the distance between the nodal point in consideration and neighbours. The relation is also motivated mathematically. Furthermore, next to predicting the rate at which cancer progresses, the calculations suffer from uncertainty from multiple sources. The first source of uncertainty is the probabilistic nature of the model itself, and the second source of uncertainty comes from poor knowledge regarding the values of the input parameters. These uncertainties make that each simulation only represents one of the very many possible scenarios. Since any of these possible scenarios could describe the progression of skin cancer in a patient, we are less interested in predicting the rate at which cancer grows than in predicting the likelihood that cancer reaches a certain size within a predefined timeframe. Note that the development of cancer is not the same as the initiation of cancer. The current modelling framework is used to simulate the progression of cancer that already initiated earlier. Hence when we speak of the development of cancer, we mean the growth of earlier initiated cancer. The issue of uncertainty regarding the growth of cancer is dealt with in the current paper using Monte Carlo simulations. This probabilistic approach is innovative in this class of modelling.

Summarised, this paper contains the following key innovations:

- development of the lattice-based Markov chain model, with a mathematically sound justification of the inter-nodal transition probability based on the states of immediate neighbours;
- phenomenological incorporation of the effects of long term exposure to UV-radiation;
- evaluation of the likelihood that the cancer develops to a predefined state within a certain time-interval.

The paper is organised as follows: in Sect. 2, we formulate the physical and biological hypotheses of the model. Furthermore, some mathematical justification is given regarding the assumptions. Section 3 deals with the numerical approach of the Markov chain process and the uncertainty quantification on the basis of Monte Carlo simulations. Subsequently, we present the results in Sect. 4. Sections 5 and 6 end with a discussion and conclusions regarding the modelling framework and results.

## The mathematical model

First we describe the model and motivate the choices that we make mathematically. We consider a domain $D \subset {\mathbb {R}}^3$ that is divided into a set of *N* nodal points ${\mathcal {N}} = \{1,\ldots ,n\}$, in which each node *i* has position $\mathbf{x}_i = (x_i,y_i,z_i)$. All positions are fixed in time *t*. From a biological perspective, these nodes may correspond to single cells, or to a cluster of cells, or even a spatial part of the body. For each of these nodes, we consider its set of neighbours, indicated by ${\mathcal {N}}_i$, which is mathematically defined by

$$\begin{aligned} {\mathcal {N}}_i = \{j \in {\mathcal {N}}~ |~ j \hbox { is a nearest neighbour node of node } i\}. \end{aligned}$$

Only nearest neighbours are incorporated to prevent huge computational burdens. Each node can be in two states: either in ‘cancer state’ or in ‘non-cancer state’. This is indicated by the binary switch parameter $S_i$:

1 $$\begin{aligned} S_i = {\left\{ \begin{array}{ll} 0, &{} \text {node }i\text { is in `non-cancer state';} \\ 1, &{} \text {node }i\text { is in `cancer-state'}. \end{array}\right. } \end{aligned}$$

This implies that if $S_i = 0$ for all $i \in {\mathcal {N}}$ then the tissue is absolutely free of cancer, whereas if $S_i = 1$ for all $i \in {\mathcal {N}}$ then the tissue is completely cancerous. All other situations are intermediate states. Next, we assume that each node, say node *i*, that is in ‘non-cancer state’ has a likelihood to change state to ‘cancer state’. This likelihood satisfies an (memory-less) exponential probability distribution with probability rate $\lambda _i$, that is, we have the following probability distribution

2 $$\begin{aligned} f(s,\lambda _i) = \lambda _i \exp (-\lambda _i(s) (s-t)), \text { for } s \ge t. \end{aligned}$$

Hence over a time interval of length $\tau$, we have the following likelihood for transition from state $\{S_i = 0\}$ to state $\{S_i = 1\}$:

3 $$\begin{aligned}&P(S_i(t + \tau ) = 1 | S_i(t) =0)) = \int _t^{t+\tau } \lambda _i(s) \exp (-\lambda _i(s) (s-t))ds \nonumber \\&\quad \approx 1 - \exp (-\lambda _i(t) \tau ) = \lambda _i \tau + {\mathcal {O}}(\lambda _i \tau )^2. \end{aligned}$$

Irreversibility of the transition implies $P(S_i(t + \tau ) = 0 | S_i(t) =1)) = 0$. The total likelihoods are given by

4 $$\begin{aligned} P(S_i(t+\tau ) = 0)= & {} P(S_i(t+\tau )=0|S_i(t) = 0) P(S_i(t) = 0) \nonumber \\&+ P(S_i(t+\tau )=0|S_i(t) = 1) P(S_i(t) = 1), \nonumber \\ P(S_i(t+\tau ) = 1)= & {} P(S_i(t+\tau )=1|S_i(t) = 0) P(S_i(t) = 0) \nonumber \\&+ P(S_i(t+\tau )=1|S_i(t) = 1) P(S_i(t) = 1), \end{aligned}$$

and hence we have the following probability matrix over time-interval $\tau$:

5 $$\begin{aligned} \mathbf{P}_{\tau }= & {} \begin{pmatrix} P(S_i(t+\tau ) = 0 | S_i(t) = 0) &{}&{} P(S_i(t+\tau ) = 0 | S_i(t) = 1) \\ \\ P(S_i(t+\tau ) = 1 | S_i(t) = 0) &{}&{} P(S_i(t+\tau ) = 1| S_i(t) = 1) \end{pmatrix} \nonumber \\= & {} \begin{pmatrix} 1 - \int _t^{t+\tau } \lambda _i(s) \exp (-\lambda _i(s) (s-t))ds &{}&{} 0 \\ \\ \int _t^{t+\tau } \lambda _i(s) \exp (-\lambda _i(s) (s-t))ds &{}&{} 1 \end{pmatrix} \approx \begin{pmatrix} 1 - \lambda (t) \tau &{}&{} 0 \\ \\ \lambda (t) \tau &{}&{} 1 \end{pmatrix}. \end{aligned}$$

This matrix can be used to simulate the dynamics of the fraction of cancerous regions over time, by recurrently computing over time points $\tau ,\ldots ,k \tau$

6 $$\begin{aligned} \mathbf{y}_{(k+1)\tau } = \mathbf{P}_{\tau } \mathbf{y}_{k \tau }, \end{aligned}$$

where the first and second component of $\mathbf{y}_{k \tau }$ represents the average fraction of nodes that are in ‘non cancer state’ and ‘cancer state’ respectively.

Solving the average fraction in ‘cancer state’, that is the second component of $\mathbf{y}_{k\tau }$, which we call $p(k \tau )$, from Eq. (6), gives

7 $$\begin{aligned} p(k \tau )= & {} 1 - \prod _{j=1}^k \left( 1 - \int _{(k-1)\tau }^{k \tau } \exp (-\lambda (s) (s-(k-1)\tau ) ds\right) \approx 1 \nonumber \\&- \prod _{j=1}^k \left( 1 - \lambda ((j-1)\tau ) \tau \right) . \end{aligned}$$

The fraction $p(k\tau )$ can also be interpreted as the likelihood that a node is in ‘cancer state’. From** Eq. (7), we get

8 $$\begin{aligned} \begin{array}{ll} \displaystyle { p(k \tau ) - p((k-1)\tau ) = 1 - \prod _{j=1}^k \left( 1 - \lambda ((j-1)\tau ) \tau \right) - \left[ 1 - \prod _{j=1}^{k-1} \left( 1 - \lambda ((j-1)\tau ) \tau \right) \right] } \\ \\ \quad \displaystyle {=\prod _{j=1}^{k-1} \left( 1 - \lambda ((j-1)\tau ) \tau \right) - \prod _{j=1}^k \left( 1 - \lambda ((j-1)\tau ) \tau \right) } \\ \\ \quad \displaystyle {=\prod _{j=1}^{k-1} \left( 1 - \lambda ((j-1)\tau ) \tau \right) (1 - (1 - \lambda ((k-1)\tau ) \tau ))} \\ \displaystyle {\quad = (1 - p((k-1)\tau )) \lambda ((k-1)\tau ) \tau .} \end{array} \end{aligned}$$

Dividing the above equation by $\tau$, gives

9 $$\begin{aligned} \frac{p(k \tau ) - p((k-1)\tau )}{\tau } = (1 - p((k-1)\tau )) \lambda ((k-1)\tau ). \end{aligned}$$

Taking the limit $\tau \rightarrow 0$ (and sending $k \rightarrow \infty$), upon $(k-1)\tau = t$, we get

10 $$\begin{aligned} p'(t) = (1 - p(t)) \lambda (t). \end{aligned}$$

With $p(0)= 0$, this gives

11 $$\begin{aligned} p(t) = 1 - \exp \left( -\int _0^t \lambda (s)ds\right) . \end{aligned}$$

### Proposition 2.1

Let $$P_{\tau } = \begin{pmatrix} 1 - \int _{t}^{t+\tau } \lambda (s) \exp (-\lambda (s)(s-t) )ds &{} 0 \\ \int _{t}^{t+\tau } \lambda (s) \exp (-\lambda (s)(s-t)) ds &{} 1 \end{pmatrix}, $$ and let $r(t) = 1 - p(t)$, then

12 $$\begin{aligned} \begin{array}{ll} \begin{pmatrix} r'(t) \\ p'(t) \end{pmatrix} = \begin{pmatrix} -\lambda (t) &{} 0 \\ \lambda (t) &{} 0 \end{pmatrix} \begin{pmatrix} r(t) \\ p(t) \end{pmatrix}, \end{array} \end{aligned}$$

implying

13 $$\begin{aligned} r(t)= & {} \exp (-\int _0^t \lambda (s) ds), \nonumber \\ p(t)= & {} 1 - \exp (-\int _0^t \lambda (s) ds). \end{aligned}$$

### Proof

From the hypothesis, it follows that

14 $$\begin{aligned} \begin{array}{ll} r(t+\tau ) = (1 - \int _{t}^{t+\tau } \lambda (s) \exp (-\lambda (s)(s-t) )ds) r(t), \\ \\ p(t+\tau ) = \int _{t}^{t+\tau } \lambda (s) \exp (-\lambda (s)(s-t) )ds r(t) + p(t). \end{array} \end{aligned}$$

The above equations can be rewritten as

15 $$\begin{aligned} r(t+\tau ) - r(r)= & {} - r(t) \int _{t}^{t+\tau } \lambda (s) \exp (-\lambda (s)(s-t) )ds, \nonumber \\ p(t+\tau ) - p(t)= & {} r(t) \int _{t}^{t+\tau } \lambda (s) \exp (-\lambda (s)(s-t) )ds \nonumber \\= & {} (1 - p(t)) \int _{t}^{t+\tau } \lambda (s) \exp (-\lambda (s)(s-t) )ds. \end{aligned}$$

From this, we get, from division by $\tau$, taking the limit $\tau \rightarrow 0$, and using de l”Hospital’s Rule

16 $$\begin{aligned} \begin{array}{ll} \displaystyle {r'(t) = \lim _{\tau \rightarrow 0} \frac{r(t+\tau ) - r(t)}{\tau } = - \lim _{\tau \rightarrow 0} r(t) \frac{ \int _{t}^{t+\tau } \lambda (s) \exp (-\lambda (s)(s-t) )ds}{\tau }}\\ \displaystyle {\quad = -r(t) \lambda (t)}, \\ \\ \displaystyle {p'(t) = \lim _{\tau \rightarrow 0} \frac{p(t+\tau ) - p(t)}{\tau } = \lim _{\tau \rightarrow 0} (1-p(t)) \frac{\int _{t}^{t+\tau } \lambda (s) \exp (-\lambda (s)(s-t) )ds}{\tau } } \\ \\ \displaystyle { \quad =(1 - p(t)) \lim _{\tau \rightarrow 0} \frac{\int _{t}^{t+\tau } \lambda (s) \exp (-\lambda (s)(s-t) )ds}{\tau } = (1-p(t)) \lambda (t)}. \end{array} \end{aligned}$$

From this, we get

17 $$\begin{aligned} \begin{array}{ll} r'(t) = -\lambda (t) r(t), \\ \\ p'(t) = \lambda (t) (1 - p(t)) = \lambda (t) r(t). \end{array} \end{aligned}$$

From this, we get

$$\begin{aligned} r(t) = \exp (-\int _0^t \lambda (s) ds), \\ p(t) = 1 - \exp (-\int _0^t \lambda (s) ds). \end{aligned}$$

This proves the assertion. $\square$

Since the expected number of nodes in cancer satisfies a Markov chain process, see Eq. (6) and the above relation, the convergence to the steady-state of full cancer takes place asymptotically as $t \rightarrow \infty$ (for $\lambda > 0$). In other words if *p*(*t*) denotes the probability that a node is in ‘cancer state’ at time *t*, then $\displaystyle {\mathrm{lim}_{t \rightarrow \infty } p(t) = 1}$, whereas $0< p(t) < 1$ for $t > 0$. The time to reach the state where all the nodes are in ‘cancer state’ increases with the number of nodes due to the asymptotic convergence. For this reason, we only monitor the minimal time at which half of the nodes are in cancer state, that is $f = 1/2$, in the Monte Carlo simulations to avoid unnecessarily large computing times. It is easy to see that the dynamic system will converge to the state $S_i = 1$ for all $i \in {\mathcal {N}}$ as $t \rightarrow \infty$ ($k \rightarrow \infty$) if $\lambda _i(t) > 0$ for $t > 0$ for all $i \in {\mathcal {N}}$.

It is well known that cancer progresses by mutation of healthy cells, proliferation of cancer cells and by migration of cancer cells. Cell migration proceeds by several mechanisms, such as taxis processes (migration towards stiffer tissue regions (durotaxis), chemotaxis (migration towards higher concentration of oxygen or nutrients)) or as a result of random walk, see for instance Lo et al. (2000). This is the reason why regions that are affected by cancer, that is, they are in ‘cancer state’, contaminate healthy regions. For simplicity, we consider two nodes on the *x*-axis, with coordinates $x_L= 0$ and $x_R = 1$. Let $x_L$ be in ‘cancer state’ while $x_R$ is not in ‘cancer state’. Let $\lambda$ be the probability rate, from the exponential distribution, for $x_R$ to change to the ‘cancer state’ due to $x_L$ being in ‘cancer state’. Then the expected length of the time-interval to change to the ‘cancer state’ for node $x_R$ is given by

$$\begin{aligned} {\mathbb {E}}(t) ={\hat{t}} = \frac{1}{\lambda }. \end{aligned}$$

Suppose now that between node $x_L$ and $x_R$, we have the following rearrangement of nodal points: $x_j = x_L + j h$, $x_n = x_R$ and $n h = 1$, where all these nodes, except $x_0 = x_L$ are not in the ‘cancer state’. Let $\lambda ^{(h)}$ be the probability rate parameter to change node $x_j$ to the ‘cancer state’, given that one of its neighbours at a distance *h* was in ‘cancer state’, then the expected length of the time interval to change to the ‘cancer state’ is given by

$$\begin{aligned} {\hat{t}}_j = \frac{1}{\lambda ^{(h)}}, \end{aligned}$$

in node *j*. Note that this considers the expected length of time interval to transform a node at a distance *h* from a node that was in ‘cancer state’. The total expected value for the length of the time interval to change all nodes into ‘cancer state’ should be equal to the expected time to change node $x_R$ to the ‘cancer state’ and hence we have

18 $$\begin{aligned} \sum _{j=1}^n {\hat{t}}_j = \frac{n}{\lambda ^{(h)}} = {\hat{t}} = \frac{1}{\lambda }. \end{aligned}$$

Herewith, we get

19 $$\begin{aligned} \lambda ^{(h)} = n \lambda = \frac{\lambda }{h}, \end{aligned}$$

which gives the probability rate between two nodes of complementary states separated by a distance *h*. From the above relation, it can be observed that if $h \rightarrow 0$, then $\lambda ^{(h)}$ tends to infinity, which implies that the expected time for the transition is given by

$$\begin{aligned} \lim _{h \rightarrow 0} {\mathbb {E}}(t) = \lim _{h \rightarrow 0} {\hat{t}}_j = 0, \end{aligned}$$

which means that the transition occurs immediately almost surely as $h \rightarrow 0$. We summarise the overall conclusion in Proposition 2.2.

### Proposition 2.2

Consider $\mathbf{x}_L$ and $\mathbf{x}_R$ and let $||\mathbf{x}_L - \mathbf{x}_R|| = 1$. Suppose that at time *t* one of the two points is in ‘cancer state’, whereas the other point is not in ‘cancer state’. Let $\lambda$ be the probability rate from the exponential distribution to transfer the nodal point that is not in ‘cancer state’ to the ‘cancer state’, then for two points $\mathbf{x}_A$ and $\mathbf{x}_B$, separated by distance $||\mathbf{x}_A - \mathbf{x}_B|| = h$, where exactly one of the two points is in ‘cancer state’, the probability rate to transfer the other point to the ‘cancer state’ is given by

$$\begin{aligned} \lambda ^{(h)} = \frac{\lambda }{h}. \end{aligned}$$

This probability rate is consistent in expected value of the length of the transformation time interval. In a more-dimensional setting, a node that is not in ‘cancer state’ could have multiple neighbours that are in ‘cancer state’. Consider node *j* that is not in ‘cancer state’. Suppose that *k* of its nearest neighbours are in ‘cancer state’. For each node in ‘cancer state’, its neighbour that is not in ‘cancer state’, say node *j*, has a likelihood of

20 $$\begin{aligned} p = \int _t^{t + \tau } \lambda ^{(h)} \exp (-\lambda ^{(h)} (s - t)) ds = 1 - e^{-\lambda ^{(h)} \tau }, \end{aligned}$$

to change to the ‘cancer state’ during time interval $\tau$. Hence the likelihood that this neighbour will not change its state is equal to $P(S_j(t + \tau )=0 | S_j(t) = 0) = 1-p$. Consider node *j* having *k* neighbours that are in ‘cancer state’ at time *t*, then

$$\begin{aligned} P(S_j(t+\tau )=0|S_j(t) =0) = (1 - p)^k. \end{aligned}$$

This implies that

$$\begin{aligned} P(S_j(t+\tau ) = 1|S_j(t) = 0) = 1 - (1 - p)^k. \end{aligned}$$

As long as $p \ll 1$, we have

$$\begin{aligned} P(S_j(t+\tau ) = 1|S_j(t) = 0) \approx k p. \end{aligned}$$

Using the exponential distribution, see Eq. 20, we arrive at

$$\begin{aligned} P(S_j(t+\tau ) = 1|S_j(t) = 0) = 1 - (1 - p)^k = 1 - e^{-k \lambda ^{(h)} \tau } = 1 - \exp {\left( -\frac{k}{h}\lambda \tau \right) }. \end{aligned}$$

Therefore, next to the distance of the node to the neighbours that are in ‘cancer state’, the probability rate $\lambda _i = \frac{k}{h}\lambda$ depends on the number of neighbouring nodes that are in ‘cancer state’. In order to be able to deal with various spatial rearrangements and grids, we generalise the formalism to having neighbouring nodes that have different separations in Proposition 2.3:

### Proposition 2.3

Consider nodal point $\mathbf{x}_k$, which is not in ‘cancer state’ at time *t*, with nearest neighbouring nodal points ${\mathcal {N}}_k$, that are, respectively, separated from $\mathbf{x}_k$ by distances $d(\mathbf{x}_k,\mathbf{x}_{j}) = ||\mathbf{x}_k - \mathbf{x}_{j} ||$, $j \in {\mathcal {N}}_k$. Let $\lambda$ be the probability rate in the exponential distribution to change a nodal point to the ‘cancer state’ due to a nearest neighbour at unit distance. Then

21 $$\begin{aligned} \lambda _k = \lambda \sum _{j \in {\mathcal {N}}_k} \frac{S_{j}(t)}{d(\mathbf{x}_k,\mathbf{x}_j)}, \end{aligned}$$

where $\lambda _k$ represents the probability rate in the exponential distribution for the transition of nodal point $\mathbf{x}_k$.

### Proof

Consider nodal point $\mathbf{x}_k$, which is not in ‘cancer state’, and only consider its neighbours $\{\mathbf{x}_{kq}\}$ that are in ‘cancer state’, hence $q \in {\mathcal {N}}_k^\textit{can} := \{q \in {\mathcal {N}}_k: S_q(t) = 1\}$. Let $p_{qk}$ be the probability that nodal point *q* contaminates nodal point *k* to the ‘cancer state’ during time interval $\tau$. Then from Proposition 2.2, we have

22 $$\begin{aligned} p_{qk} = 1 - \exp \left( {-\frac{\lambda \tau }{d(\mathbf{x}_k,\mathbf{x}_q)}}\right) . \end{aligned}$$

The probability that nodal point $\mathbf{x}_k$ remains in ‘non cancer state’ is given by

23 $$\begin{aligned} P(S_k(t+\tau ) = 0 | S_k(t) = 0) = \prod _{q \in {\mathcal {N}}_k^\textit{can}} (1 - p_{qk}), \end{aligned}$$

where we account for the fact that each neighbour $\mathbf{x}_{kq}$, $q \in {\mathcal {N}}_k^\textit{can}$ may send $\mathbf{x}_k$ to the ‘cancer state’. Using the above equation, Proposition 2.2, and Eq. (20), we get

24 $$\begin{aligned} \begin{array}{ll} \displaystyle { P(S_k(t + \tau )=1|S_k(t)=0) = 1 - P(S_k(t + \tau )=0|S_k(t)=0) }\\ \\ \displaystyle { \qquad =1 - \prod _{q \in {\mathcal {N}}_k^\textit{can}} \exp \left( -\frac{\lambda \tau }{d(\mathbf{x}_k,\mathbf{x}_q)}\right) = 1 - \exp \left( -\lambda \tau \sum _{q \in {\mathcal {N}}_k^\textit{can}}\frac{1}{d(\mathbf{x}_k,\mathbf{x}_q)}\right) .} \end{array} \end{aligned}$$

The definition of ${\mathcal {N}}_k^\textit{can}$ and $S_q(t)$, then gives

25 $$\begin{aligned} P(S_k(t + \tau )=1|S_k(t)=0) = 1 - \exp \left( -\lambda \tau \sum _{j \in {\mathcal {N}}_k}\frac{S_j(t)}{d(\mathbf{x}_k,\mathbf{x}_j)}\right) . \end{aligned}$$

Hence

$$\begin{aligned} \lambda _k = \lambda \sum _{j \in {\mathcal {N}}_k} \frac{S_{j}(t)}{d(\mathbf{x}_k,\mathbf{x}_j)}. \end{aligned}$$

This proves the assertion. $\square$

The above result in Proposition 2.3 does not yet take into account any UV radiation. To model the effect from exposure to UV radiation, we extend the above equation with a term

26 $$\begin{aligned} \lambda _i = \lambda \sum _{j \in {\mathcal {N}}_i} \frac{S_{j}}{d(\mathbf{x}_i,\mathbf{x}_j)} + \alpha R_i, \end{aligned}$$

where $R_i$ denotes the life time burden UV intensity that is experienced by node *i*, and the $\alpha$–parameter accounts for the sensitivity of cell *i* with respect to UV-radiation. Hence summarised, the model is based on the hypothesis that cancer progresses to node *i*, as a result of the following three factors:

1. the number of nearest neighbours of node *i* in the ‘cancer state’;
2. the distance between node *i* and nearest neighbours that are in the ‘cancer state’;
3. the life burden intensity of UV light that is experienced by node *i*.

## Numerical implementation

First we explain how we apply the model in the final calculations where we simulate with the full model. For all nodes $i \in {\mathcal {N}}$, we initially impose $S_i = 0$. Then at each time-step, the light intensity $R_i$ is computed on all the nodes. Subsequently, a sample from the exponential distribution with probability rate $\lambda _i$ for each node is drawn over time-interval $\tau$. Based on the resulting samples, the state of each node is changed to the ‘cancer state’ or not. Subsequently the time is incremented by the time step $\tau$. This procedure is repeated with the exception that at the next time steps, we also check the state of the nearest neighbours. This whole procedure is repeated until half of the nodes are in ‘cancer state’. The fraction of nodes in ‘cancer state’, for a total of *n* nodes, is computed via

27 $$\begin{aligned} f = p_n(k \tau ) = \frac{1}{n} \cdot \displaystyle {\sum _{i \in {\mathcal {N}}}} S_{i}(k \tau ), \end{aligned}$$

at time $k \tau$. The time $T_f$, denoting the minimal time at which a certain predefined fraction of the nodes is in ‘cancer state’, *f*, is stored.

**Fig. 1:**
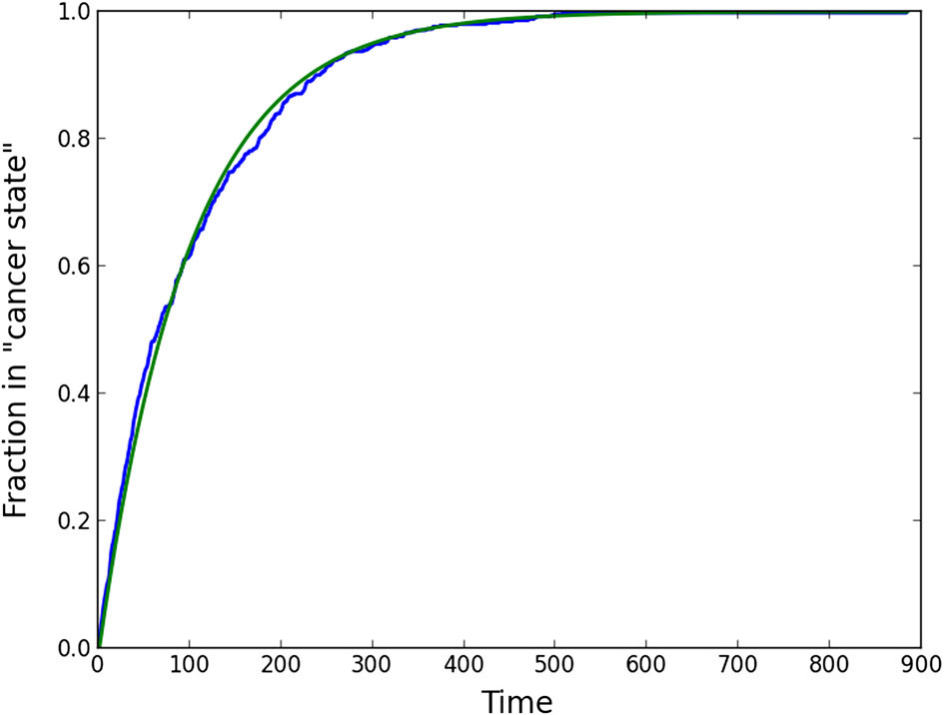
The fraction of nodes in ‘cancer state’ as a function of time. The green curve represents the deterministic result from Eq. (7), whereas the blue curve represents one sample from the stochastic model result using $8 \times 8 \times 8$ nodes with $\lambda = 0.01$ (color figure online)

In order to test the algorithm, the transition probability for each node was taken equal to $\lambda$, regardless the state of the neighbours. Then the solution should approximate the expression from Eq. (7), that is $p_n(k \tau ) \rightarrow p(k \tau )$ for $k \in {\mathbb {N}}$ as $n \rightarrow \infty$. We calculate the root mean square error ($\textit{RMS}_n$) with Eq. (7), given by

28 $$\begin{aligned} \textit{RMS}_n = \sqrt{\frac{\displaystyle {\sum \nolimits _{k=1}^{n_t} (p_n(k\tau ) - p(k \tau ))^2}}{n_t}}, \end{aligned}$$

where $n_t$ is the time index at which all the nodes have transformed to the ‘cancer state’, that is $S_j(n_t \tau ) = 1$ for all $j \in {\mathcal {N}}$. For parametric choices $\lambda = 0.01$, and a cubic domain of $1 \times 1 \times 1$, and $n = 8 \times 8 \times 8$ nodes, we show the results in Fig. 1. From this figure, the exponential growth character is obvious and further $p_n(k \tau )$ and $p(k \tau )$ only differ by a root mean square error of 0.01209. Note that Fig. 1 has only been added to illustrate graphically that the macro-Markov chain model and the lattice model are consistent.

We listed the results for the root mean squared error for one sample on various numbers of nodes in Table 1.

**Table 1:** Root mean squared errors for one sample of various numbers of nodes

Number of nodes	Root mean squared error
$8 \times 8 \times 8$	$0.1209 \times 10^{-1}$
$16 \times 16 \times 16$	$0.5086 \times 10^{-2}$
$32 \times 32 \times 32$	$0.1774 \times 10^{-2}$
$64 \times 64 \times 64$	$0.1007 \times 10^{-2}$

From these results, it is concluded that if $n = 32 \times 32 \times 32$ the sampling error is sufficiently small.

In the final computations, the Monte Carlo method is carried out by repeating the simulations 1000 times ($N = 1000$). The average minimal time at which a fraction *f* of the nodes are in ‘cancer state’ is computed by the arithmetic mean of all the sample $T_f^{(i)}$, hence by

29 $$\begin{aligned} {\hat{T}}^N_f = \frac{1}{N} \sum _{j = 1}^N T_f^{(j)}, \end{aligned}$$

where $T_{f}^{(j)}$ denotes the *j*th Monte Carlo trial of the minimal time at which a fraction of *f* of the nodes is in ‘cancer state’. These simulations are done for the full model. The above sample mean is an unbiased estimator for the expected value of the minimal time, that is ${\mathbb {E}}({\hat{T}}^N_f) = \mu _{f}$. Here $\mu _f = {\mathbb {E}}(T_f)$ denotes the expected value of the minimal time at which a fraction *f* of the nodes is in ‘cancer state’. The Monte Carlo Error (MCE) based on *N* trials (samples) is defined by

30 $$\begin{aligned} \textit{MCE}_N := \sqrt{\textit{Var}[{\hat{T}}_f^N]}, \end{aligned}$$

where $[{\hat{T}}_f^N]$ consists of *m* batches of *N* samples. The above equation requires the use of multiple batches containing a lot of simulations or the division of a Monte Carlo sequence into multiple batches. In the remainder of this section, we derive a statistically sound and practical representation of the Monte Carlo Error that can be determined using one batch of simulations only. The derivation tightly follows the footprints outlined by Koehler et al. (2009). From the Strong Law of Numbers, we have

$$\begin{aligned} {\hat{T}}_f^N \rightarrow \mu _f, \text { as } N \rightarrow \infty . \end{aligned}$$

The Central Limit Theorem implies that the above sample mean converges in distribution to a normal distribution via

31 $$\begin{aligned} \sqrt{N} ({\hat{T}}_f^N - \mu _f) \longrightarrow _d {\mathcal {N}}(0,\sigma _T^2), \text { as } N \rightarrow \infty , \end{aligned}$$

where $\sigma _T^2 = {\mathbb {E}}((T_f - \mu _f)^2)$ represents the standard deviation of $T_f$. The above relation implies

32 $$\begin{aligned} \textit{Var}[\sqrt{N}({\hat{T}}_f^N - \mu _f)] \rightarrow \sigma _T^2, \text { as } N \rightarrow \infty . \end{aligned}$$

Since $Var(\mu _f) = 0$, the above equation implies

$$\begin{aligned} \textit{Var}[\sqrt{N} {\hat{T}}_f^N] = N \textit{Var}[{\hat{T}}_f^N] \rightarrow \sigma _T^2, \text { as } N \rightarrow \infty . \end{aligned}$$

Hence we have

33 $$\begin{aligned} \textit{Var}[{\hat{T}}_f^N] \rightarrow \frac{\sigma _T^2}{N} = \frac{{\mathbb {E}}((T_f - \mu _f)^2)}{N}, \end{aligned}$$

as $N \rightarrow \infty$. The sample variance is given by

$$\begin{aligned} s_N^2 = \frac{1}{N-1} \sum _{j=1}^N (T_f^{(j)} - {\hat{T}}_f^N)^2. \end{aligned}$$

Since the sample variance is an unbiased estimator for $\sigma _T^2$ (that is ${\mathbb {E}}(s_N^2) = \sigma _N^2$), we take $\sigma _T^2 \approx s_N^2$, then the right-hand side of Eq. (33) is estimated by

34 $$\begin{aligned} \textit{Var}[{\hat{T}}_f^N] \approx \frac{s_N^2}{N}, \end{aligned}$$

Hence the MCE is estimated by

35 $$\begin{aligned} {\widehat{\textit{MCE}}}_N = \frac{s_N}{\sqrt{N}} = \sqrt{\frac{{\sum \nolimits _{j=1}^N (T_f^{(j)} - {\hat{T}}_f^N)^2}}{N(N-1)}}. \end{aligned}$$

The above equation is used for the estimation of the Monte Carlo Error. Note that the sample variance is an unbiased estimator for the variance $\sigma ^2_T$ (of $T_f$), then as a result of Jensen’s Inequality, the sample standard deviation, $s_N$, is a biased, but consistent estimator of $\sigma _T$ (that is $\displaystyle {\lim \nolimits _{N \rightarrow \infty } s_N = \sigma _T}$). Hence the above estimator for the MCE is biased, though consistent. Hence for large *N*, the above equation gives a good approximation for the MCE. Therefore, we use the above equation for the estimation of the MCE.

## Computer simulations

All simulations were done in an implementation in Python 2.7.5 on a MacBook Air with a 1.4 GHz Intel Core i5 processor and 8 GB 1600 MHz DDR3 memory. The spatial domain represents a generic area of skin. The top of the domain represents the surface of the epidermis and in which the bottom represents the basement membrane, which is a physical barrier that inhibits further progression of cancer. Further progression of cancer over the basement membrane proceeds by different biological mechanisms, such as the slower process of epithelial-mesenchymal transformation, than the ones that are considered in the current manuscript. First, we present various results of one simulation of how cancer progresses through the domain in the course of time. Subsequently, we show results from Monte Carlo simulations in two spatial dimensions, where we are interested in the length of the time interval that is needed to have 50% of the domain occupied by cancer. We will show the correlations of this length of time interval with the three input parameters.

### Time-evolution of cancer in 3D

First we show model predictions of skin cancer progression over time. In the plots that we show, we only display the nodes that are in ‘cancer state’. These nodes are indicated by red dots. The other nodes, which are not in ‘cancer state’, are not displayed for the sake of visibility in the three dimensional configuration. In the coming results, we present three different cases in three spatial dimensions in the coming subsections. We use $32 \times 32 \times 32$ nodes in the closed cubic domain $(x,y,z) \in [0,1]^3$ and $\lambda = 0.01$. Further, we kept $\alpha _i = 0$ for all $i \in {\mathcal {N}}$, which implies that cancer only progresses through nearest–neighbour interaction.

#### Progression of melanomas

**Fig. 2:**
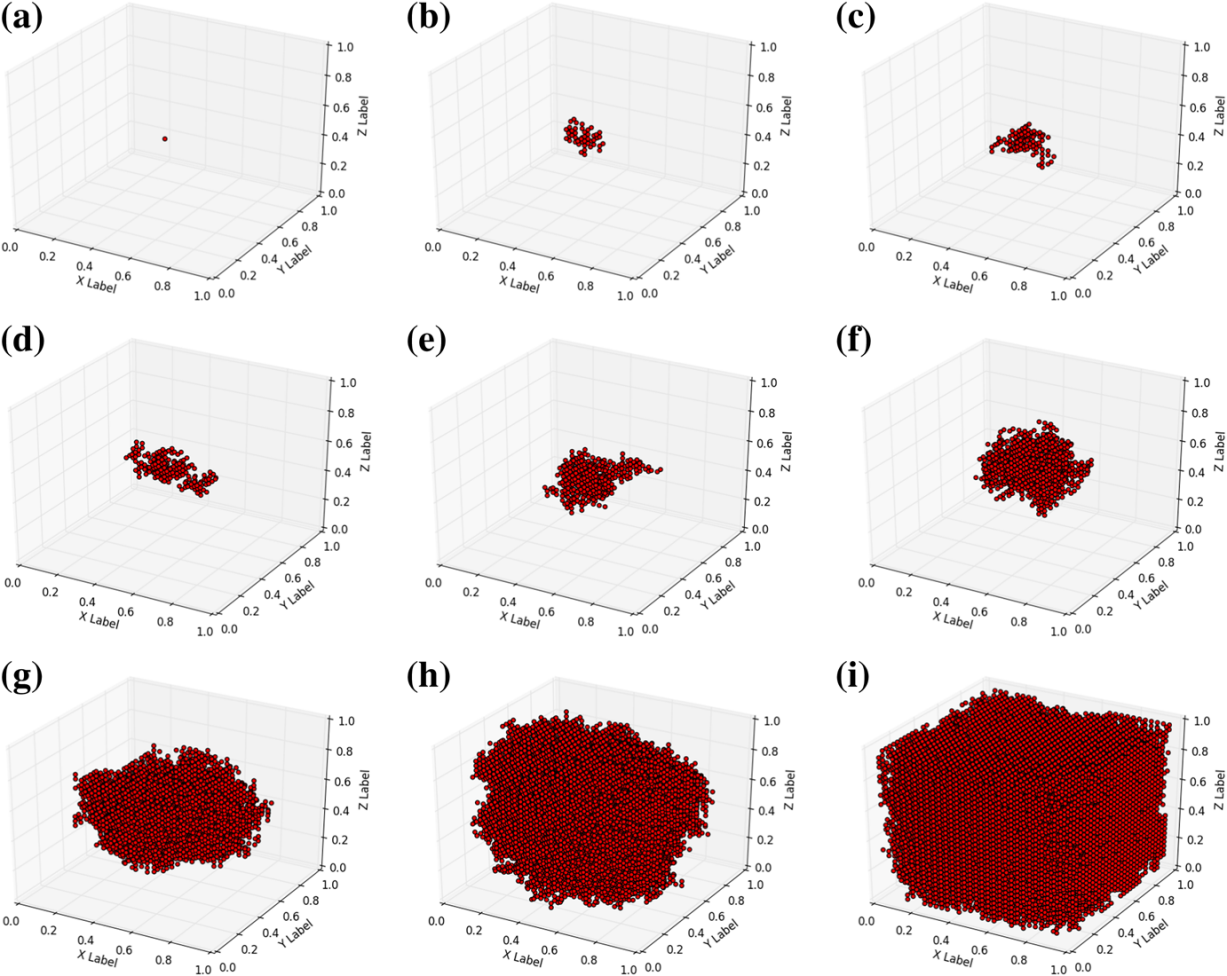
Snapshots of early stage progression of cancer from the middle of the domain of computation. Snapshots were taken at 1, 50, 100, 200, 400, 1000, 5000, 15,000 and 30,000 nodes in ‘cancer state’; Corresponding times are 0, 2.35, 3.1, 4.15, 5.3, 7.1, 11.3, 15.85, 22.3 dimensionless time units

Melanomas generally progress from mutated melanocytes, which are located on or near the basal membrane separating the epidermis from the dermis. Although these cancers are often enhanced by excessive exposure of the skin to UV-radiation (generally type B, which progresses deeper into the dermis), melanomas often develop as a result of genetics, or from moles. Therefore, we consider the case that one cell in the center of the domain ($(x,y,z) = (0.5,0.5,0.5)$), away from the skin surface, is in ‘cancer state’, which could represent a melanoma that spreads towards the top and the bottom of the skin surface. Several snapshots have been shown in Fig. 2, at the early and later stages. From the figures, it is observed how the cancer develops from the centre and spreads into locations further away. In Fig. 2, it is seen that at the later stages the boundary of the computational domain starts to influence the results. It can be seen how the model gives a possible representation of the geometrical development of the early stage cancer except at the final stages, where boundary effects start to dominate, see Fig. 2i.

**Fig. 3:**
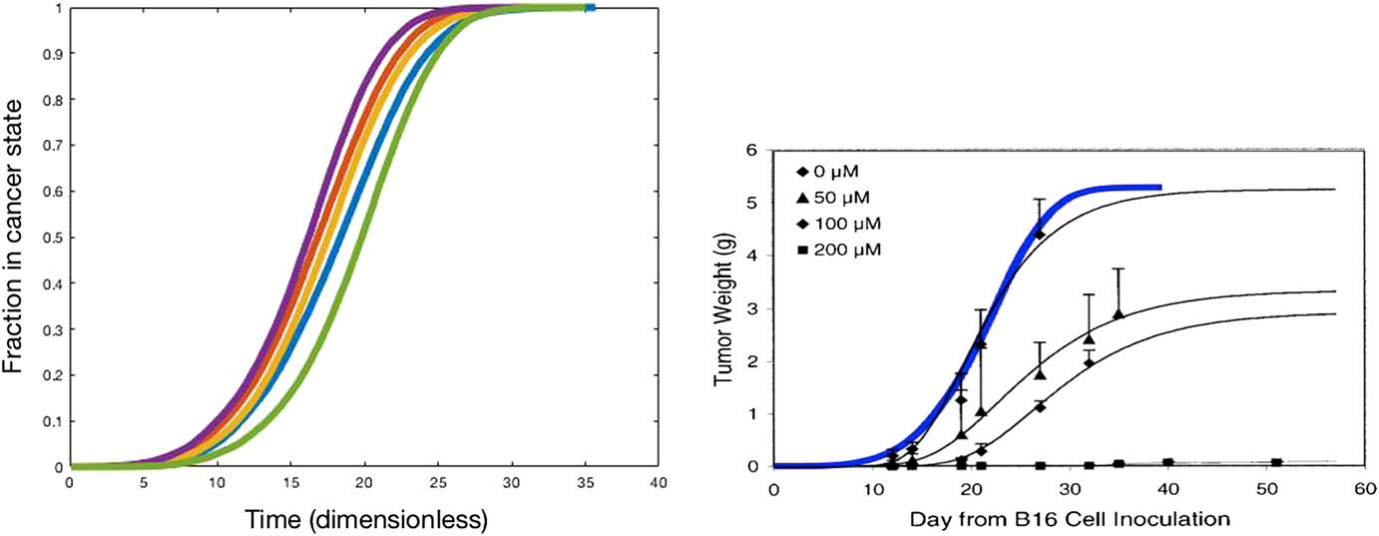
Left: five sample runs of the fraction of the nodal points that transformed into the cancer state versus time using $32 \times 32 \times 32 = 32768$ nodes; Right: a fitted result to the experiments done in Demidem et al. (2001). The thick solid blue curve right has been obtained by the use of the current spatial Markov chain model; the error bars denote the measured results by Demidem et al. (2001). The black curves are fitted results to the Gompertz model (color figure online)

In Fig. 3, we show the fraction of nodes that has transformed into the cancer state as a function of time for five different samples using identical input parameters and initially the central node at (0.5, 0.5, 0.5) was assumed to be in cancer state. The curves, which do not coincide entirely as a result of the stochastic nature of the model, all exhibit the so–called S-shape in accordance with the classical Gompertz model.

Although the experimental outcomes by Demidem et al. (2001) were obtained for mice, we managed to fit our model to their results. Their experiments measured the mass of the tumor. Assuming a mass density of water, which is given by $$\rho = 1000$$ kg/$$\hbox {m}^3$$, to represent the density of the tumor, implies that 5.3 gram of tumor, which is their final tumor mass, corresponds to a tumor volume of $$5.3 \times 10^{-6}$$$$\hbox {m}^3$$. This implies that we bound the tumor in a cubic box with edge length of $$5.3^{1/3} \times 10^{-2}$$ m. Using a bisection procedure to fit our modelling results to the Gompertz curve in Demidem et al. (2001) indicated that $$\lambda \approx 1.5 \times 10^{-4}$$$$m^{-1}~ day^{-1}$$. The result is shown in Fig. 3, where the fraction that we computed can be converted to actual mass by setting $$m_{T} = \rho \cdot f \cdot V_R$$, where *f*, $$m_T$$ and $$V_R$$, respectively, denote the volume fraction of the tumor, tumor mass and total end volume. Comparison with Fig. 1 in Demidem et al. (2001) shows a good agreement between their results and our results. Note that we fitted our model to the data with the highest values of the tumor weight in the study by Demidem et al. (2001). This sequence of data corresponds to non-drug treated melanoma. The thick solid blue curve in Fig. 3b has been obtained by the use of our spatial Markov chain model. The error bars represent the experimental results by Demidem et al. (2001), and the thin solid black curves represent the fits by Demidem et al. (2001) using the Gompertz model. This agreement demonstrates the predictive value of our model.

#### Progression from the surface: squamous basal-cell carcinomas

In the modelling, we approximate the development from the upper layers by the progression from the top. Squamous basal-cell carcinomas develop from the top, but not from the very top (since the squamous cells are dead at the very top and the surface is covered by keratin) of the skin surface (the top of the epidermis), and these cancers are known to develop as a result of a life time excessive exposure to UV-radiation. In this section, we take the enhancement of the transition from ‘non cancer state’ to ‘cancer state’ as a result of UV-radiation into account in a phenomenological way. The likelihood of transition is given by

36 $$\begin{aligned} \lambda _i = {\left\{ \begin{array}{ll} \displaystyle {\lambda \sum _{j \in {\mathcal {N}}_i} \frac{S_{j}}{d(\mathbf{x}_i,\mathbf{x}_j)} + \alpha ,} &{} z_i = 1, \\ \\ \displaystyle {\lambda \sum _{j \in {\mathcal {N}}_i} \frac{S_{j}}{d(\mathbf{x}_i,\mathbf{x}_j)},} &{} z_i < 1. \end{array}\right. } \end{aligned}$$

**Fig. 4:**
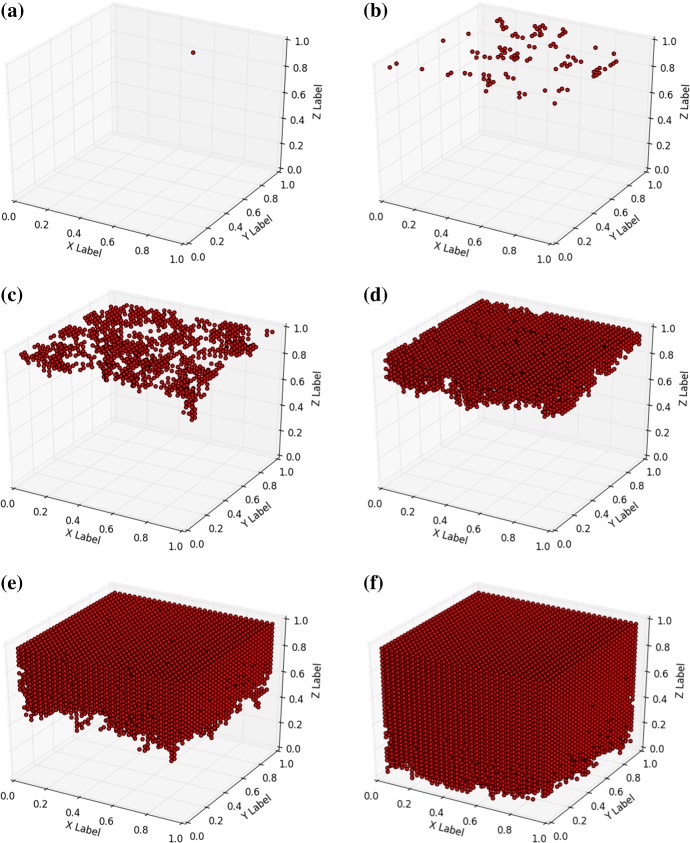
Snapshots of progression of cancer from the upper layer of the domain of computation. Snapshots were taken at 1, 100, 1000, 5000, 15,000 and 30,000 nodes in ‘cancer state’; Corresponding times are in 0, 0.8, 2.7, 6.6, 15.7 and 28.4 dimensionless time units

Here $\alpha$ takes into account the life time burden of exposure to UV-radiation, which only acts on the nodal points that are located on the top surface of the skin. In this section, we took $\lambda = 0.01$ and $\alpha = 0.1$. A small $\alpha$ value represents a relatively small life time burden of exposure to UV-radiation, whereas large values represent large portions of life time exposure to UV radiation. The life time burden of radiation is determined by the fraction of time the skin of a patient is exposed to sunlight and by the intensity of the UV radiation exposed to. Hence it makes a difference whether the skin is exposed to one hour of evening sun (low intensity) or one hour to the sun at the mid of the day (that is at noon, high intensity). Initially, all nodes are in the ‘non cancer’ state. The transition to the ‘cancer state’ is caused by the exposure to UV-radiation and in the current simulations, we assume that only the top cells are exposed to UV. Penetration of UV-radiation into deeper layers is neglected in the current run. The results are shown in Fig. 4, where it can be seen that cancer progresses from the top layer towards the bottom. At the initial stage, only nodes on the top can change state to ‘cancer state’. As time proceeds, the nearest neighbour interaction makes the cancer move towards the bottom. In practice, the use of sun cream could result into a lower value of $\alpha$, which would give a lower initiation rate (a larger incubation time) of cancer.

**Fig. 5:**
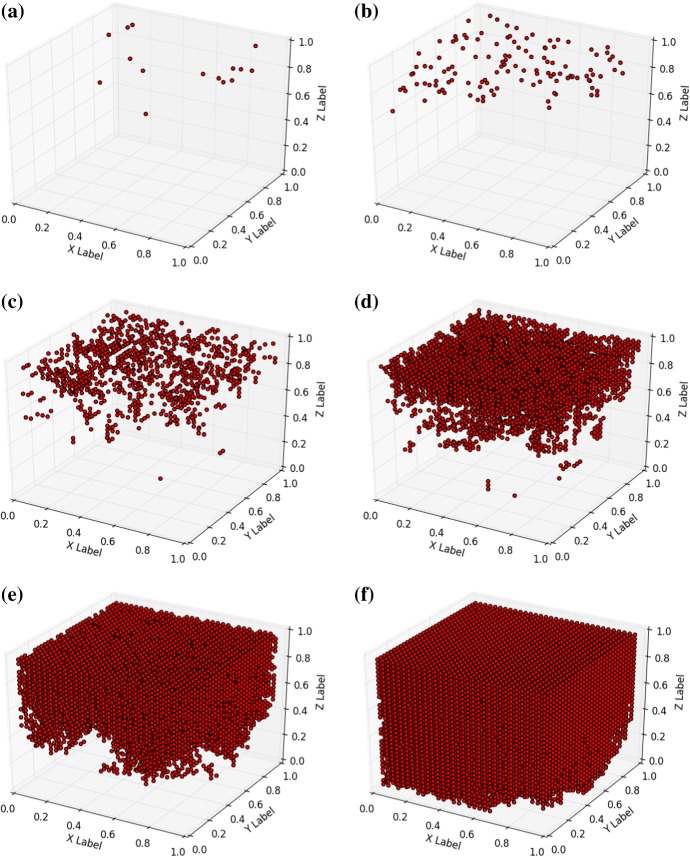
Snapshots of progression of cancer from the upper layer of the domain of computation. Snapshots were taken at 1, 100, 1000, 5000, 15,000 and 30,000 nodes in ‘cancer state’; Corresponding times are in 0, 0.3, 1.3, 2.85, 6.0 and 15.7 dimensionless time units

#### Progression and gradually decreasing initiation with the depth of the domain

Basal-cell carcinomas (BCC) generally develop from basal cells, which are predominantly located in the basal membrane. Melanomas develop from melanocytes, which are also predominantly located in the basal membrane. In order to model generic forms of skin cancer, where we incorporate the attenuation and gradual decay of the life long burden of UV-radiation into the skin, we use the following phenomenological law for UV transmission

37 $$\begin{aligned} R(z) = e^{-\beta {\hat{z}}}, \end{aligned}$$

where the top layer of skin is at the coordinate ${\hat{z}} = 0$, hence we have ${\hat{z}} = 1 - z$ related to the physical coordinate *z*. This gives the following relationship for the $\lambda$-parameter

38 $$\begin{aligned} \lambda _i = \lambda \sum _{j \in {\mathcal {N}}_i} \frac{S_{j}}{d(\mathbf{x}_i,\mathbf{x}_j)} + \alpha e^{-\beta \hat{z_j}}. \end{aligned}$$

Higher values of $\beta$ imply that the solar irradiation is absorbed faster as the UV radiation penetrates the skin. Lower values of $\alpha$ represent the case that the skin is less sensitive to the UV radiation and that cancer initiation at the top of the skin is inhibited. Application of sun cream could be modelled by larger values of $\beta$ and/or lower values of $\alpha$. Using $\alpha = 0.1$ and $\beta = 1$, we get the results from Fig. 5. Large values of $\beta$ correspond to low transmissibility of UV-radiation into the skin. Well functioning sunscreen (sun cream) will increase the $\beta$–value and hence radiation decays very fast in the top layers of skin. Thereby the probability of cancer initiation, as well as progression can be reduced. Once again, the $\alpha$-value represents the exposure of the skin surface to sunlight. Sun cream can also decrease this value due to its performance to shield the skin from UV-radiation. It can be seen that cancer develops a bit more homogeneously than in Fig. 4 in the early stages, because the probability rate $\lambda$ is distributed more homogeneously and hence more nodes that do not yet have a neighbour node that is already in ‘cancer state’ are allowed to transform into ‘cancer state’ in Fig. 5. However, at the latest stages the development of cancer becomes similar to the behaviour in Fig. 4 as a result of nearest neighbour interaction.

### Monte Carlo simulations in two dimensions

The stochastic nature of the model requires a statistical assessment of the model results. First, we assess the scalability of the model with respect to consistency of the results in terms of convergence of the results as the grid size tends to zero. Secondly, we deal with the consequences from uncertainties in the model parameters.

#### Scalability of the model

The probabilistic nature of the model requires the assessment of its scalability. From adjusting the probability rates according to the number of immediate neighbours in ‘cancer state’ and the inter-nodal distance, it is to be expected that the expected time to reach the state in which half of the nodes are in ‘cancer state’, that is, ${\hat{T}}_{0.5}$ does not depend on the spatial resolution *h*. Therefore, we carried out multiple simulations with various values of spatial resolution in a one-and two dimensional setting using $N = 1000$ samples of Monte Carlo simulations. The results have been listed in Table 2. It can be seen that for the larger stepsizes *h* (the smaller resolutions), there is still a dependence, but that as *h* becomes smaller, then the results become less dependent on *h*.

Note that these simulations contain an error as a result of the spatial resolution, and as a result of the number of Monte Carlo samples.

**Table 2:** Dependence on the internodal distance *h*

Number of nodes per dimension	${\hat{T}}_{0.5}$ in ${\mathbb {R}}^1$	${\hat{T}}_{0.5}$ in ${\mathbb {R}}^2$
25	0.4495	0.2955
50	0.4155	0.2525
100	0.4076	0.2328
200	0.4075	0.2190
400	0.4058	0.2111

#### Implications from uncertainties in the data

The simulations so far are realisations of stochastic processes like in the case of the transition probability. Next to the uncertainty in transition, the actual values of input data such as the transition likelihood, as well as the sensitivity with respect to UV-radiation and the amount of UV radiation suffer from uncertainty. Therefore, we vary all these parameters using normal distributions and we carry out Monte Carlo simulations using the sampling values. From the simulations, we compute the expected value of the time that the cancer has grown to a predefined volume fraction in the tissue, as well as the probability distribution of the time that the cancer has grown to a predefined volume fraction. This allows the estimation of the likelihood that cancer develops to a predefined volume fraction within a certain amount of time. To this extent, we use the following sampling for the input parameters $\lambda$, $\alpha$ and $\beta$:

39 $$\begin{aligned} \begin{array}{lll} \lambda \sim {\mathcal {N}}(\mu _{\lambda },\sigma ^2_{\lambda }),&\alpha \sim {\mathcal {N}}(\mu _{\alpha },\sigma ^2_{\alpha }),&\beta \sim {\mathcal {N}}(\mu _{\beta },\sigma ^2_{\beta }) \end{array} \end{aligned}$$

**Table 3:** Values of the input parameters

Quantity	Mean	Variance
$\lambda$	0.005	0.001
$\alpha$	0.1	0.02
$\beta$	3	0.6

**Fig. 6:**
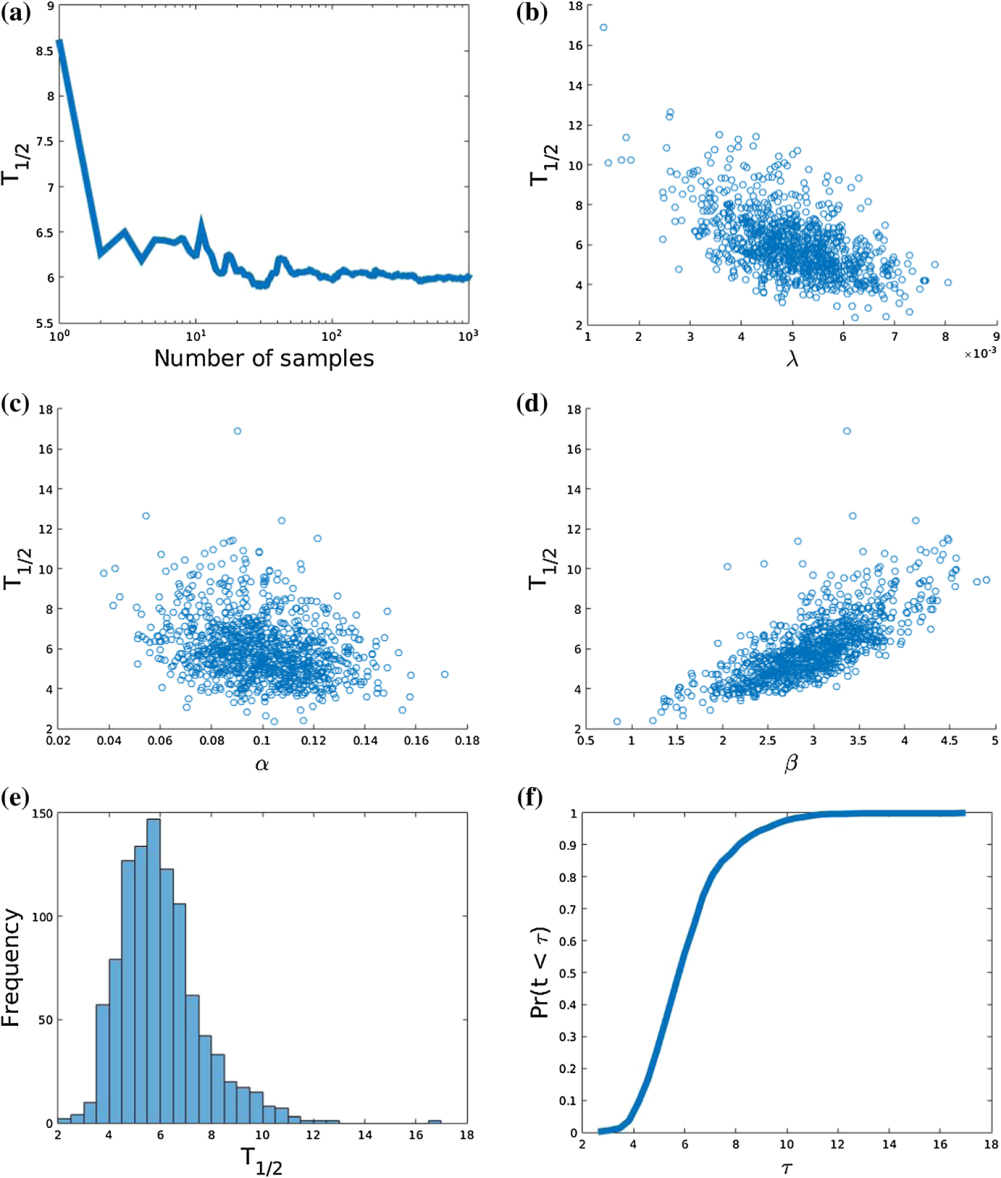
**a** Convergence of the Monte Carlo simulations; **b** scatter plot between $\lambda$ and $T_{1/2}$; **c** scatter plot between $\alpha$ and $T_{1/2}$; **d** scatter plot between $\beta$ and $T_{1/2}$; **e** histogram of outcomes of $T_{1/2}$; **f** cumulative probability that $T_{1/2} \le \tau$

The values of the above statistical distributions have been listed in Table 3, which still only contain hypothetical values. The results of the Monte Carlo simulations for $100 \times 100$ nodes are displayed in Fig. 6. It can be seen that $N = 1000$ simulations gave good convergence of $T_{1/2}$ with respect to the Monte Carlo simulations. This is confirmed by the Monte Carlo Error (MCE), which can be estimated consistently using the estimator in Eq. (35) $\textit{MCE}_N \approx 0.0505$. Further, the scatter plots between $T_{1/2}$ and $\lambda$, $\alpha$ and $\beta$ have been shown as well, from which it can be been seen that $T_{1/2}$ tends to decrease with increasing $\lambda$ and $\alpha$ (to a smaller extent). An increase is observed with $\beta$. Note that $\beta$ represents the amount of decay of UV-radiation through the skin. Using the Pearson correlation test, the correlations with their respective *p*-values have been listed in Table 4. These numbers confirm the slight negative correlation between $T_{1/2}$ and $\alpha$ and a stronger negative correlation between $T_{1/2}$ and $\lambda$. A somewhat stronger positive correlation is observed between $T_{1/2}$ and $\beta$. Hence cancer is more likely to progress for larger values of $\lambda$ and larger values of $\alpha$, which, respectively, represent the proliferation rate (including cancer cell division and cancer cell migration), and the intensity (or exposure) of UV-radiation. Cancer indeed progresses less quickly for larger UV-absorption (decay) by the skin. These predictions are in line with intuition and common observations and hence in this sense the model provides a reasonable description of the phenomenon. Next to the scatter plots, we present the histogram representing the frequency of occurrence of $T_{1/2}$. It can be seen that $T_{1/2}$ is not normally distributed with some kurtosis with a tail for large values of $T_{1/2}$. Using the data, we compute the cumulative likelihood that $T_{1/2}$ is at most equal to a certain value. That is, we compute

40 $$\begin{aligned} Pr(T_{1/2} \le T) = F_N(T) = \frac{1}{N} \sum _{j=1}^N {\mathbb {I}}_{[0,T]} (T_{1/2}^{(j)}). \end{aligned}$$

where $T_{1/2}^{(j)}$ denotes the *j*th sample, and ${\mathbb {I}}_{[0,T]}(x)$ denotes the indicator function, which is characterised by

$$\begin{aligned} {\mathbb {I}}_{[0,T]}(x) = {\left\{ \begin{array}{ll} 1, &{} \text {if } x \in [0,T], \\ 0, &{} \text {else}. \end{array}\right. } \end{aligned}$$

The results have been plotted in Fig. 6. The cumulative probability should be interpreted as the likelihood that the tissue is seriously infected by cancer within a specific time interval. We finally remark that the uncertainty analysis could also be applied to Eq. (26), to get a statistical distribution of the $\lambda$–parameter such that one could sample from this obtained distribution. This has been omitted in the present study.

**Table 4:** Correlation coefficients with $T_{1/2}$

Quantity	Correlation	*p* value
$\lambda$	$-$ 0.5502	$< 0.0001$
$\alpha$	$-$ 0.2827	$< 0.0001$
$\beta$	0.7389	$<0.0001$

## Discussion

We propose a phenomenological model for the development of skin cancer. The model is very generic and could be applied to different types of cancers as well. The formalism is a two-state (binary) model and it is based on transition probabilities, which reflects the likelihood that a certain portion of the domain changes state. Hence the occurrence of the transition from the ‘non cancer state’ to the ‘cancer state’ depends on the states of the points surrounding the point of consideration. It is assumed that the change of state is irreversible. A strong point of the current model is that it is very generic, simple to understand, and that it contains only few steering parameters. Further, the model is easy to implement in more-dimensional settings. This facilitates a straightforward parameter sensitivity analysis. The current implementation further allows a three-dimensional evaluation of the geometry of the tumor. Demidem et al. (2001) experimentally investigate tumor growth in mice under the influence of drug treatments. Their measured tumor (melanoma) mass as a function of time is fitted to the classical Gompertz model, which displays an S-shape (see Figures 1, 3 and 4 in Demidem et al. 2001). This S-shape is also reproduced by our model, see Fig. 3. Furthermore, our model also quantitatively matches with the results in Demidem et al. (2001) if the right input values are chosen.

The current phenomenological model is used to simulate the progression of cancer in the epidermis as long as it does not cross the basement membrane. The process of crossing the basement membrane proceeds by different biological mechanisms. However, if the rate of progression of the cancer across the basement membrane is known then one can model this phenomenon by adjusting the transition probability rate over this barrier. In this way one can model further metastasis by using a location (and if necessary time-dependent) dependent transition probability $\lambda$. This has been omitted in the current paper.

The model has a stochastic nature and this implies that each modelling outcome represents a *possible* scenario. Formally, using a time-step and a finite number of points, implies that the number of possibilities, that is the number of scenarios, is finite and hence the set of possible outcomes, $\varOmega$, is finite although in a continuous time-setting the number of possible outcomes is infinite. In this context one should mention that choosing a smaller time-step and a higher grid resolution enlarges the set of all possible outcomes, and the set of possible outcomes may become arbitrarily large if the numerical resolution is increased. Next to the uncertainty as a result of the stochastic nature of the model, the values of the input parameters are not known. This lack of information provides an additional source of uncertainty. If their mean and standard deviation is known as well as their statistical distribution, then samples of these values can be inserted into the stochastic model and herewith one can compute the likelihood of different scenarios. This is what we have done in the current paper and this enables us to estimate the probability that certain scenarios will happen. These scenarios are for instance the time-span needed for the cancer to have invaded half of the domain. This is done by the use of Monte Carlo simulations in the current work and this approach to estimate the likelihood of a scenario is not yet that common in the field of mathematical biology. Since for an accurate prediction, many Monte Carlo samples are needed, these computations are relatively expensive. One could benefit from a more clever application of Monte Carlo techniques by using a Multi-Level technique, which is based on taking Monte Carlo samples at various grid resolutions. It is possible to prove that the number of Monte Carlo simulations that are needed decrease with increasing resolution. More information about Multi Level Monte Carlo techniques can be found in Cliffe et al. (2011). Since the setting of the current model differs from the deterministic model that is dealt with in Cliffe et al. (2011), the investigation of Multi Level Monte Carlo techniques to this type of models in the future is of great interest.

In the current simulations, we did not know any values of input parameters, hence no quantitative conclusions can be drawn from our simulations regarding the development of skin cancer in humans. Hence a calibration with experimental or clinical outcomes remains to be done in future studies, though we demonstrated by the results of Demidem et al. (2001) and Fig. 3 that such a calibration is indeed feasible. In this paper we are using a general approach to simulate any skin cancer. Different types of skin cancer can be taken into account when the initial computational domain is created. Each of the major skin cancer types is connected to certain cell types. If the initial skin structure contains squamous, basal and melanocyte cells in the epidermal layer of skin and fibroblast, macrophage, and adipocyte cells in dermal layer, it is possible to take into account skin cancer type specific modelling progression. We additionally remark that the present model can also be applied to modelling progression of other types of cancers.

Regarding alternative computational models for skin cancer, we note the studies by Poleszczuk and Enderling (2013), Morais et al. (2017), Gallinaro et al. (2013) and Ciarletta et al. (2001). In Poleszczuk and Enderling (2013), a cellular-automata framework for tumor growth in general has been developed. In their model, each cancer cell occupies a single gridnode of the cellular automata mesh. Cancer cells are distinguished into cancer stem cells and non-stem cancer cells. The first type is immortal and has an unlimited proliferation potential, whereas the second type is mortal with a finite proliferation potential. Cell division proceeds in an asymmetric way, where the likelihood that a cancer stem cell divides into a cancer stem cell is not the same as the probability of division into a non-stem cancer cell. The model explicitly incorporates the migration and division of cells. These features make their model more sophisticated than our current model, where only a Markov-chain process is used with a simple transition probability, taken into account the number of occupied (in cancer state) neighbours. In our model, each grid node does not necessarily coincide with a cellular position, but with a centre of a control volume which is in either cancer state or in non cancer state. Furthermore, our model incorporates the exposure to life burden UV-radiation by adjusting the probability of transition probability from one node to another. Although our own model and the model by Poleszczuk and Enderling (2013) contain some similarity by using a cellular automata model with stochastic principles, the main difference is that their model takes into account more biological processes explicitly, whereas the same biological processes are incorporated implicitly in the transition probability in the current formalism in our model. Hence our own model contains fewer parameters, which makes the quantification of uncertainty easier. The formalism by Morais et al. (2017) models the relation between the proliferation of melanocytes and contact inhibition. The two-dimensional framework models a mono-layered cell culture, where the Widom-Rowlinson model from statistical physics is used. Two different cell types are considered: cancer cells and healthy cells. In their lattice model, each lattice vertex (or node) is occupied by one cell only. In order to simulate contact inhibition, they use an exclusion principle to bound the distance between cells of different type or of the same type from below. Their principle allows to incorporate that tumour-tumour cells are more allelophilic than tumour-healthy cells or healthy-healthy cells. Cells are more allelophilic if their minimal exclusion distance is smaller, which means that they are allowed to be located closer to each other. This model, like in the study of Poleszczuk and Enderling (2013), is based active migration and division of cells using stochastic processes. The model by Morais et al. (2017) is interesting in the sense that it incorporates more biological processes, like contact inhibition, it still is a somewhat more complicated model than our formalism with a larger number of parameters that are difficult to get from literature or measurements. Gallinaro et al. (2013) model melanoma cell (melanocyte) migration by the use of an elastic continuum. Their approach is based on a system of ordinary differential equation to describe the displacement of the spatial coordinate in the tissue in a one-dimensional configuration. Their model contains some similarities with the morpho-elastic models, which were applied in Koppenol and Vermolen (2017) and Menon et al. (2017). The approach is entirely deterministic and one-dimensional, contrary to our current model which is entirely stochastic and three-dimensional. Ciarletta et al. (2001) consider a partial differential equations-based model for the radial growth of melanoma. Their approach is based on mixture theory, where they incorporate various phases by accounting for their volume fractions. These different phases are melanoma cells, interstitial liquid, and basal laminae. Furthermore, a balance of momentum, where inertial effects are neglected, result into a saddle-point like system (to be compared with Stokes’ flow or Biot’s poroelasticity model) for the velocity vectors of the various components and their pressures. This framework is combined with a balance equation for the nutrients. For the movement of the interface between the tumor and non-tumor regions, a free boundary condition is imposed. Their simulations suggest instability (analogous to a Mullins-Sekerka instability) of the interface with respect to long-wavelength perturbations. Their approach is interesting in the sense that the mechanisms are dealt with in a physics-based way. However, the amount of input parameters, such as for the application of the interaction forces, makes the model prone to uncertainty since the values of the input parameters are often hard, or even impossible, to get. Furthermore, the numerical approximation of the solution to the saddle point problem is often quite challenging. The application of uncertainty quantification on their model will be much more complicated and expensive from a computational point of view than the uncertainty quantification on our own cellular automata based model.

An important weakness of the current formalism is the oversimplification of the description of the mechanisms governing the biological process. The mechanisms involve cell division (proliferation), cell mutation, and cell migration, but also the interaction of cells of different phenotypes through mechanical and chemical signals. All these processes have been simplified through the transition probability, which represents the progression of cancer from one point to another. Though we proved how the transition probability changes with the distance between adjacent points and number of neighbouring points that are in the cancer state, it still simplifies all the mechanisms behind it. Since each model represents a description of a certain process or phenomenon from the modeller’s point of view, it is very hard, and probably even impossible, to find a model that describes such a phenomenon perfectly. However, currently we aim at formulating a *simple* and *qualitatively useful* model that describes possible scenarios of the development of skin cancer. A clear advantage of such a simple model is its tractability and having just few parameters that are to be tuned or varied for the sake of uncertainty quantification. A disadvantage is that it does not contain much of the underlying mechanisms of the phenomenon studied by us. We could have used, and we did this in different studies, more complicated models that entail many of the mechanisms. These models give more complicated mathematical problems with more input parameters which need to be determined through experiments. Having such a complicated model, also yields a larger dimension of the parameter space and hence the uncertainty of such a model raises the legitimate question whether a more *realistic* model gives a better and more accurate description than a simple model such as the current model. If only prediction is what one is aiming at, then, it is less important that the model takes into account many of the underlying mechanisms and for these purposes a less sophisticated model could be useful as well. Furthermore, as it has been said earlier, the complicated models often involve complex mathematical problems that need to be solved by using computationally expensive procedures so that the parameter sensitivity analysis and/or prediction of likelihood of particular scenarios becomes very expensive and may even become unpractical if not enough computational power is available.

More complicated models could be based on continuum-scale models based on partial differential equations, where one takes into account chemical and mechanical signals. This amounts to solving elliptic, hyperbolic and parabolic partial differential equations. An example is given in Ciarletta et al. (2001) where the radial growth of melanoma is modelled. Here one can use finite-element strategies for the approximation of the solution to the partial differential equations in case of more-dimensional cases. The equations are then solved for displacements, stresses, concentration of chemicals and cell densities. One can track the cell density of cancer cells to get an impression of the development of cancer.

It is known that skin cancer initiation predominantly takes place on or near the basement membrane, where cells are most proliferative. At the very top of the epidermis, the cells are keratinized or even dead and hence cancer is not likely to initiate there. In this view the simulations in Figs. 4 and 5 should be considered as academic cases, which have limited biological relevance. The objective of the current paper is to show the applicability of the model, which can be calibrated to obtain an agreement with the experiments by Demidem et al. (2001), as well as showing the straightforward quantification of uncertainty using the current model.

As it has been mentioned earlier, the current model has a very generic nature. Since the progression and development of (viral) infections is based on similar features of changing state from a ‘non-infected state’ to an ‘infected state’ depending on the states of neighbouring points, we think that our model is also applicable to modelling the infection of tissues by viruses.

We finally remark that we will compare the current model to Fisher-Kolmogorov’s partial differential equation (see Murray 2004; Kaliappan 1984), in which the solution can be interpreted as the probability that a particular point in space is in cancer state at a certain time.

## Conclusions

We developed a spatial Markov chain model for the progression of cancer in skin tissue under the influence of proliferation rate, UV-radiation intensity, and absorption rate by the skin. The model has been evaluated using Monte Carlo simulations, which allow the establishment of correlation between the cancer development rate and the input parameters like cancer proliferation rate, UV radiation intensity and the UV absorption coefficient by the skin tissue. Furthermore, the model allows the computation of the likelihood that a tissue becomes seriously infected by cancer within a specific time-interval. We additionally demonstrated by computations that it is possible to fit our model to experiments where the tumor mass is evaluated over time. In future studies, we want to use real-world valued parameter values by calibration to experiments on humans, as well as relating $\lambda$ to the migration rate of travelling waves in the context of partial differential equations like Fisher-Kolmogorov’s equation.
